# Harmonizing the Gut Microbiome and Cellular Immunotherapies: The Next Leap in Cancer Treatment

**DOI:** 10.3390/cells14100708

**Published:** 2025-05-14

**Authors:** Kendall Harrington, Khalid Shah

**Affiliations:** 1 Center for Stem Cell and Translational Immunotherapy, Brigham and Women’s Hospital, Harvard Medical School, Boston, MA 02115, USA; kharrington21@bwh.harvard.edu; 2 Department of Neurosurgery, Brigham and Women’s Hospital, Harvard Medical School, Boston, MA 02115, USA; 3 Harvard Stem Cell Institute, Harvard University, Cambridge, MA 02138, USA

**Keywords:** cellular therapy, T cells, mesenchymal stem cells, natural killer cells, gut microbiome, cancer, antibiotics, probiotics, short-chain fatty amino acids

## Abstract

The gut microbiome, a diverse community of microorganisms, plays a key role in shaping the host’s immune system and modulating cancer therapies. Emerging evidence highlights its critical influence on the efficacy and toxicity of cell-based immunotherapies, including chimeric antigen receptor T cell, natural killer cell, and stem cell therapies. This review explores the interplay between gut microbiota and cellular immunotherapies, focusing on mechanisms by which microbial metabolites and microbial composition impact treatment outcomes. Furthermore, we discuss strategies to leverage the gut microbiome to optimize therapeutic efficacy and minimize adverse effects. A deeper understanding of the relationship between the gut microbiome and cellular immunotherapies can pave the way for more effective cell-based therapies for cancer.

## 1. Current Landscape of the Gut Microbiome and Immunotherapies

The human microbiome is a vast collection of commensal bacteria, archaea, fungi, and viruses in and on the body [1]. The human intestine alone contains nearly 30 trillion bacteria, representing over 400 different species [2]. Bacteria compose the majority of the gut microbiome, with less than 8% belonging to viruses, archaea, and other eukaryotes [3]. Historically, the role of the gut microbiota in human health and disease was poorly understood, especially regarding the impact of viruses, archaea, and other non-bacterial eukaryotes on homeostasis and host functions, including immune responses. However, with the advent of novel molecular techniques and the use of gnotobiotic mice, broader roles of the gut microbiota have been unraveled. In particular, the microbiota has been shown to substantially influence the immune system’s development. Specific bacterial species have been shown to influence distinct immune cell populations, contributing to either anti- or pro-inflammatory responses [4,5,6]. The gut microbiota is also known to affect the tumor microenvironment through microbial metabolites, which can suppress anti-tumor activity and enhance tumor proliferation, or enhance immune surveillance and support tumor cell killing [7]. As a result, there is increased exploration into the role of microbiota in cancer development and therapy.

Immunotherapy represents a transformative approach in cancer treatment that utilizes the body’s immune system to fight cancer. In recent years, immune checkpoint inhibitor (ICI) therapy targeting programmed cell death protein 1 (PD-1) and cytotoxic T-lymphocyte–associated antigen 4 (CTLA-4) has considerably advanced treatment across a range of cancers [8]. Multiple studies have elucidated the relationship between ICI efficacy and microbiota, and certain phyla have been linked to differential outcomes post-ICI treatment in solid tumors [9,10,11,12,13]. For example, an increase in bacterial phyla *Firmicutes* and *Verrucomicrobia* populations has a proven correlation with better ICI outcomes, while an increase in *Proteobacteria* is linked to poor response to ICI treatment [14]. Similarly, another gut bacterial genus, *Bifidobacterium*, plays an important role in response to immunotherapy, as populations of this genus are linked with delayed tumor growth, better T cell infiltration of tumors, and improved response to anti-PD1 therapy [9,11,12,15].

Cell-based immunotherapies are emerging as the next generation of immunotherapies for cancer. Although substantial work has been undertaken to understand the influence of the gut microbiome on ICIs in cancer treatment, its influence on cell-based immunotherapies remains largely unexplored. Despite differences in therapeutic composition and delivery, both ICIs and cellular immuno-therapeutics share similar immune mediated mechanisms. The known link between ICIs and microbiota can be leveraged to explore this relationship in cell-based immunotherapies.

This review outlines the current interaction between the gut microbiome and cellular immuno-therapeutics for cancer. Recent pre-clinical and clinical studies on cell-based immunotherapies for cancer indicate a strong correlation between the gut microbiome and therapeutic efficacy, patient survival rates, and treatment-associated toxicities. While a definitive causal role for the microbiota has yet to be established, early evidence indicates that certain bacterial taxa affect therapeutic outcomes, and microbiota alteration could impact treatment efficacy. Building on this foundation, we explore several future outlooks for manipulating and optimizing the gut microbiome as a tool for increased therapeutic efficacy and safety of cellular immunotherapies.

## 2. Cell-Based Immunotherapies and Influential Gut Bacteria Populations

Cells used in cancer therapies are broadly categorized into autologous or allogeneic types depending on their source [16,17,18,19,20]. Autologous cells are directly derived from the patient, whereas allogeneic cells are obtained from healthy donors. Autologous therapies have a decreased chance of a reactive immune response but are more time-consuming and expensive, as the treatment is tailored to each patient. On the other hand, allogeneic therapies are more standardized for use across multiple patients. However, there is a higher chance of an immune incompatibility and rejection [16,17,18,19,20]. Both approaches possess distinct advantages and limitations, and a balanced development in both platforms of cellular therapeutics is essential for advancing the fight against cancer. Autologous cell therapies primarily include gene-edited and engineered chimeric antigen receptor T cells (CAR-T) and natural killer cells (NK cells), whereas allogeneic cellular therapies include hematopoietic stem cell transplantation (HSCT) and mesenchymal stem cells (MSCs).

CAR-T cell therapy has yielded promising results in treatment across various cancers, particularly hematologic cancers. This approach involves isolating patients’ T cells and modifying them to express a chimeric antigen receptor (CAR) on their surface. This CAR is designed to recognize proteins on the surface of cancer cells, enabling targeted immune-mediated killing [16,18]. Emerging evidence suggests that the gut microbiome significantly influences CAR-T therapy outcomes. Several bacterial genera in the microbiota, such as *Bacteroides*, *Ruminococcus*, *Eubacterium*, *Akkermansia*, and *Faecalibacterium*, are strongly linked to CAR-T efficacy in lymphoma, leukemia, and multiple myeloma [15,21,22]. In particular, *Bifidobacterium* was strongly associated with improved six-month survival post-treatment, and *Faecalibacterium* was associated with a higher complete response at day 100 post-treatment, while species in *Akkermansia* and *Ruminococcus* were linked to increased progression-free survival in patients with hematologic malignancies like multiple myeloma, leukemia, and lymphoma [15,22]. Studies that are more recent have shown a correlation between certain bacterial species, such as *Bacteroides*, and the response to CAR-T therapy [23]. Other bacterial species, including *Enterococcus*, have been found in substantially disrupted microbiomes and linked to increased toxicity, such as cytokine release syndrome [23].

Hematopoietic stem cell transplantation (HSCT) is a widely used immunotherapeutic approach for treating hematologic malignancies. Most HSCT procedures are allogeneic, which can enhance graft-versus-tumor effects but also carry a risk of immune complications. The role of the gut microbiome in the efficacy of HSCT therapies is still in its infancy. Studies have shown that leukemia patients have less diverse microbiota compared to healthy individuals, which is linked with increased mortality and domination by single taxa [24]. Notably, a more diverse intestinal microbiota was linked to lower mortality during neutrophil engraftment in patients with acute leukemia undergoing allogeneic HSCT [24]. However, regardless of initial diversity, patients commonly experience a marked loss of microbial diversity post-HSCT treatment [24,25]. Similarly, patients with hematologic malignancies who had a complete response to CAR-T therapy had more microbiome diversity than those who had a partial response [22]. Conversely, in another study, all lymphoma and leukemia patients were shown to have decreased microbiota alpha diversity prior to treatment compared to healthy adults [21].

In addition to influencing treatment efficacy, the gut microbiome can aid in quicker recovery post-treatment with HSCT. As donor stem cells begin reconstituting the immune system by generating white blood cells, T cells, NK cells, and other immune components, the composition of the gut microbiota can significantly affect this immune reconstitution. Graft-versus-host disease (GVHD) is a major side effect of HSCT that can cause illness and death [24]. In a study involving 37 children with leukemia, a higher abundance of *Ruminocacaceae* was seen in patients with quick NK and B cell reconstitution, as well as mild or no GVHD. In contrast, reduced levels of NK cells and B cells post-HSCT are associated with a lower overall survival [25]. Additionally, *Faecalibacterium* showed a strong positive correlation with B and NK cell counts [25]. Both *Ruminocacaceae* and *Lachnospiraceae*, members of the phylum *Firmicutes*, were associated with faster NK and B cell growth, lower GVHD severity, and increased overall survival mediated by the production of anti-inflammatory metabolites, such as butyrate [25]. Thus, enhancing the abundance of these bacteria may support faster immune reconstitution, reduce adverse effects, and improve overall treatment outcomes following HSCT.

Mesenchymal stem cells (MSCs) have been used in cancer immunotherapy, particularly in the treatment of solid tumors, due to their inherent ability to migrate to tumors. In pre-clinical studies of colitis-associated colon cancer (CAC), mice treated with MSCs were shown to have fewer tumors post-MSC treatment compared to untreated controls [26]. Moreover, the administration of MSCs regulated gut microbiota dysbiosis and restored gut microbiome imbalance by altering the diversity of the gut microbiome [26,27,28,29,30]. This re-balancing of the gut microbiome was related to an abundance of *Acetatifactor*, a butyrate-producing bacterium within the order *Clostridiales*, which played a key role in decreased colitis in MSC-treated mice [26]. Recent studies have further supported the correlation between MSC treatment and an increase in butyrate-producing bacteria, underscoring a potential mechanism by which MSCs promote intestinal and systemic immune homeostasis [30]. Beyond their microbiome-modulating effects, MSCs have also been shown to greatly influence the immune system by affecting T cells, NK cells, dendritic cells, and B cells [31]. Given their dual capacity to regulate both immune response and the gut microbiome, MSCs have the potential to serve as valuable players to boost the efficacy of other types of cellular immunotherapies.

NK cells play a pivotal role in innate immunity by detecting and eliminating cells under stress, like tumor cells, without the need for prior activation. Upon activation, NK cells secrete cytokines, like interferon-γ (IFNγ), which modulate the activity of other immune cells [32]. Recent studies have highlighted the direct connection between *Bacteroidetes ovatus* and NK cell activation and tumor-killing efficiency [33]. Similarly, the bacteria from the *Lactobacillus* genus have been shown to increase NK cell infiltration into the tumor microenvironment, with clinical data correlating this with improved overall survival in cancer patients [34].

The gut microbiome plays a crucial role in indirectly modulating the effectiveness of all cellular immunotherapies discussed above. Specific bacterial species, like *Faecalibacterium* and *Firmicutes*, have been linked to improved treatment outcomes through increased efficacy and reduced toxicity. With new findings determining a causal relationship between microbiota species and cellular immunotherapy efficacy, there is growing interest in leveraging microbiome manipulation to optimize treatment efficacy and minimize adverse effects. These findings highlight the importance of the gut microbiome as a key factor in treatment outcomes and suggest that future approaches may benefit from microbiome manipulation to optimize cellular immunotherapy treatment efficacy and reduce side effects.

## 3. Short-Chain Fatty Acids (SCFAs) and Other Metabolites: How Bacteria Affect Cellular Immunotherapies

Short-chain fatty acids (SCFAs), primarily acetate, propionate, and butyrate, are produced as fermentation byproducts by gut bacteria via the breakdown of dietary fibers [35,36]. Previous research has established a clear, significant relationship between microbiota-derived SCFAs and the immune system [7,37,38,39]. However, the relationship between these SCFAs and cellular immunotherapies has only recently been explored. SCFAs act as histone deacetylase (HDAC) inhibitors, inducing epigenetic changes in cells [40]. Butyrate producing bacterial populations, notably within *Clostridiales* and *Firmicutes* phyla such as *Ruminococcus* and *Faecalibacterium*, have been associated with cancer immunotherapy outcomes [21,40]. SCFAs reduce the expression of certain pro-inflammatory cytokines via butyrate, acetate, and propionate [41]. Notably, higher levels of SCFAs have been linked with increased populations of *Faecalibacterium*, *Ruminococcus*, *Bifidobacterium*, *Bacteroides*, and *Akkermansia* in multiple myeloma patients experiencing complete remission post-immunotherapy treatment [42,43]. Moreover, higher levels of butyrate have been shown to decrease Treg differentiation [21], which is significant given that Tregs suppress anti-tumor immune response and impair CAR-T cell therapy. SCFAs have also been shown to enhance CAR-T cell efficacy by increasing their tumor-killing ability through metabolic and epigenetic reprogramming [40].

NK cells are influenced by metabolites produced by microbiota [44]. SCFAs have bifunctional utility: reducing the secretion of IL-10, an anti-inflammatory cytokine that promotes tumor cell proliferation via immunosuppression, while also increasing NK extracellular vesicle secretion of IFNγ that aids in tumor-killing [41]. Recent studies have shown that butyrate, acetate, and propionate enhance the antitumor cytotoxicity of NK cells [41]. In particular, butyrate specifically increases NK cell proliferation and is positively associated with a better response in vitro in a progressive non-Hodgkin’s lymphoma cell line [41].

SCFAs also enhance IFNγ-mediated responses and T cell differentiation [41,45], contributing to stronger CD8+ T cell mediated tumor clearance [45]. Additionally, IFNγ induces the expression of TRAIL in astrocytes, which hinderss inflammation in the central nervous system (CNS) by inducing T cell apoptosis in pro-inflammatory T cells. These findings point out the impact of microbiota-derived metabolites on NK cells, playing a key role in CNS homeostasis [44]. In particular, *Akkermansia* has been shown to enhance IFNγ secretion from both CD4+ and CD8+ T cells [15]. Microbiota-derived SCFAs also have a substantial effect on immunoregulatory functions and impact the secretome of NK cells, indirectly influencing their functions [41].

**Figure 1:**
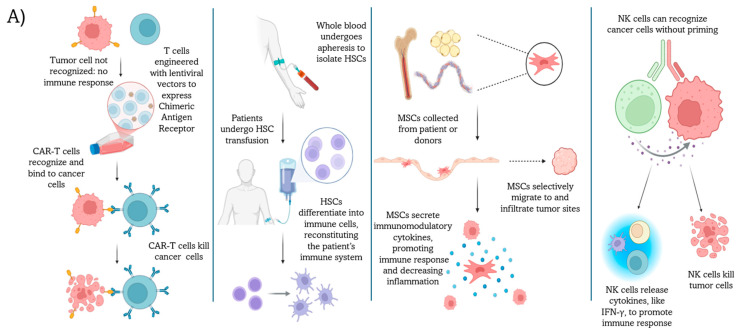

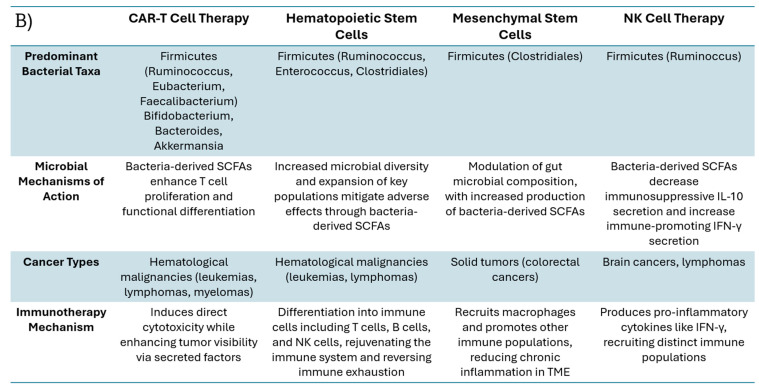
Cell-based immunotherapies and their relationship with the gut microbiome. (**A**) A schematic representation of four major cellular immunotherapies for cancer treatment. **Left** panel: CAR-T cell therapy involves modifying T cells with chimeric antigen receptors to recognize and kill cancer cells. **Middle-left** panel: HSCT therapy involves apheresis to isolate HSCs, followed by transfusion to revitalize the patient’s immune system. **Middle-right** panel: MSC therapy utilizes MSCs to promote an immune response at tumor sites. **Right** panel: NK cell therapy leverages NK cells’ innate ability to kill cancer cells unprovoked, releasing cytokines to enhance overall immune response. Created in Biorender. Shah, K. (2025) https://BioRender.com/swui7ob. (**B**) Comparison of key gut microbiota associated with each type of cell-based immunotherapy analyzed, including key bacterial taxa, their roles in therapeutic efficacy, current cancer types treated, and immunotherapeutic mechanisms for CAR-T, HSCT, MSC, and NK cell therapies.

Microbiota-derived SCFAs play a critical role in enhancing the efficacy of cellular immunotherapies. Butyrate, acetate, and propionate can modulate immune responses and enhance therapeutic efficacy, specifically for NK cells. While their role in other cell-based immunotherapies remains less explored, SCFAs consistently correlate with improved therapeutic outcomes. Their multifaceted influence on immune cell activation, differentiation, and cytokine secretion positions them as promising adjuncts in future immunotherapeutic strategies. As the field progresses, integrating microbiota-derived SCFAs into therapeutic strategies could significantly enhance the effectiveness of cancer treatments (Figure 1).

## 4. The Role of Antibiotics in Cell-Based Immunotherapies

Standard cancer treatments, like chemotherapy and radiation therapy, are often accompanied by antibiotics due to the immunosuppressive effects of these treatments, which place patients at a higher risk for infection. While the antibiotics are effective in preventing and fighting off infections by killing harmful bacteria, they also inadvertently eliminate beneficial bacteria in the gut microbiome [46,47]. The use of antibiotics, like vancomycin, has been shown to decrease the alpha diversity of the microbiota substantially [21]. This includes a diminished abundance of *Faecalibacterium*, *Eubacterium*, *Bifidobacterium*, and *Ruminococcus*, leading to lower production of SCFAs [43,48]. There is already strong evidence showing a negative association between antibiotic use and therapeutic response to ICIs due to a decrease in key influential bacteria [9,14]. Similarly, patients receiving antibiotics within three to four weeks of CAR-T treatment exhibit worse overall survival, increased neurotoxicity, and increased cancer progression [15,21,43]. In patients with lymphoma treated with anti-CD19 CAR-T therapy, those who received antibiotics had a higher relapse rate compared to those who did not receive antibiotics [43]. Exposure to broad-spectrum antibiotics prior to CAR-T treatment was associated with worse survival outcomes. Specifically, these patients exhibited substantial gut microbiome dysbiosis, resulting in a significant decrease in microbiota-derived SCFAs [43].

Importantly, certain types of antibiotics are correlated with better overall survival and CAR-T therapeutic efficacy than other types of antibiotics [21]. The use of high-risk antibiotics (HRAs), classified as those that target several anaerobic bacteria populations in the gut microbiome and result in substantially reduced microbial diversity, has been associated with significantly higher rates of disease progression compared to low-risk antibiotics [43], which have similar progression rates compared to no antibiotic exposure [15]. This distinction has been observed in HCST. For example, vancomycin use in HCST patients has been associated with lower levels of *Ruminocacaceae* and increased *Enterococcus* abundance, both markers of gut dysbiosis and SCFA deficiency [25]. Conversely, other antibiotics, like ceftazidime, were positively associated with key bacteria within *Clostridiales*, but still decreased alpha diversity [25]. An increased *Enterococcus* commonly associated with antibiotic use has been inversely correlated with SCFA levels and negatively impacts CAR-T therapy [43].

Although promising, patients treated with CAR-T immunotherapy can develop CAR-related toxicities, like cytokine release syndrome (CRS), which causes systemic inflammation, and immune effector cell-associated neurotoxicity syndrome (ICANS) [21]. In patients with non-Hodgkin’s lymphoma, increased ICANS was associated with antibiotic use within 30 days of treatment, regardless of the type of CAR-T treatment used [21]. Similarly, in HSCT, loss of microbiome diversity is often driven by antibiotic exposure and linked to GVHD related mortality. Enterococcus overgrowth, a common outcome of dysbiosis, was consistently observed across treatment centers and associated with increased GVHD risk [24]. A more diverse microbiota before, during, and after HSCT was associated with a lower risk of death and lower GVHD-related mortality [24]. Moreover, a loss of *Clostridiales*, critical butyrate producers, is associated with GVHD [25]. Antibiotics, including vancomycin, have also been shown to reduce *Clostridiales* and are linked to GVHD development [25]. Although antibiotics play a vital role in preventing infections during immunosuppressive cancer treatments, they have detrimental effects on the gut microbiota. In some cases, the microbial disruptions have been observed up to four years post-treatment [49]. Antibiotics reduce microbial diversity and deplete key species, worsening overall survival, decreasing SCFA levels, and leading to more severe side effects.

## 5. Future Perspectives

Given the rapid advancement of cellular immunotherapies for cancer treatment and the role of gut microbiota in disease development and therapy, a thorough understanding of the relationship between the two has the potential to pave the way for personalized and more effective cell-based therapies for cancer. As most new cancer treatments, particularly in Phase I and II trials, are given in tandem with standard-of-care cancer treatments, like chemo and radiation therapies, optimization of the gut microbiome is even more essential to cancer treatment. Current cancer treatments ravage the microbiota through heavy antibiotic use, causing dysbiosis. Across all types of cell-based immunotherapies, a more diverse microbiota and increased populations of key bacteria positively impacted treatment outcomes and patient responses. More specifically, MSCs have the potential to enhance the microbiota prior to treatment, leading to a more successful outcome across different cell-based therapies. By increasing microbial diversity and boosting populations of beneficial bacteria, particularly species in *Ruminocacaceae*, *Faecalibacterium*, *Clostridiales*, *Bacteroides*, *Bifidobacterium*, and *Akkermansia*, the efficacy of cell-based immunotherapies could be significantly improved.

MSCs could substantially alter the gut microbiome by increasing alpha diversity and important bacterial populations, which could heavily influence other types of cell-based immunotherapies [26,27,28,29]. This could increase SCFA production, boosting NK and mature T cells. Additionally, altered SCFA production due to gut dysbiosis has been associated with cancer progression [41]. By reducing gut dysbiosis, MSCs could limit negative side effects like GVHD in stem cell therapies and CRS in CAR-T therapy, as well as make them more effective.

Across the four types of cell-based immunotherapies analyzed, there is an obvious overlap in bacterial phyla and treatment outcomes. *Firmicutes*, specifically, play an important role in all treatment effectiveness. While there have been no established causal relationships between the gut microbiome and these therapies, there is a strong correlation between *Firmicutes* and all cell-based immunotherapies discussed here. This is likely due to the production of SCFAs by bacteria in this phylum, most notably butyrate. Future treatment options could leverage butyrate and other SCFAs for better results, potentially supplementing additional SCFAs through postbiotics to patients undergoing treatment. The gut microbiome can be analyzed in patients to determine the efficacy and side effects of various cell-based immunotherapies. Large, diverse populations of *Ruminococcus*, *Faecalibacterium*, and *Clostridiales* found in patients would suggest that treatment could be more effective with decreased toxicity. Levels of SCFAs, like butyrate, should also be considered when determining the efficacy of a treatment for patients. Pre-treatment screening of the gut microbiome in patients could be used to predict patient outcomes and support the use of one immunotherapy over another. It should be noted that several parameters affect the composition of the gut microbiota, including genetics, sex, age, BMI, diet, alcohol intake, and drug consumption. Future research should investigate how these parameters modulate the gut microbiome and, in turn, affect cellular immunotherapy outcomes.

Further studies are required to establish a definitive, causal relationship between the gut microbiome and the efficacy of cell-based immunotherapies. SCFAs produced in the microbiota are of particular interest due to their seemingly across-the-board positive role in cell-based immunotherapies. Additionally, the role of other metabolites produced by the gut microbiome needs to be understood, as it is improbable that one metabolite solely affects therapeutic efficacy. Additional research is required to identify and understand other microbiota-derived compounds that influence immune responses and therapeutic efficacy, many of which are also altered by antibiotic use [43]. 

Moreover, a deeper understanding of how specific classes of antibiotics affect cell-based immunotherapies is critical. Preclinical and early-phase clinical studies should explore this in greater depth. This could lead to more strategic, targeted antibiotic use that preserves microbial diversity and key taxa, thereby enhancing cellular immunotherapy outcomes. Currently, the type and severity of antibiotic usage could serve as a predictor for the efficacy of cellular immunotherapies in patients. The drastic impact of antibiotics on treatment outcomes leaves the door open for the future implementation of probiotics and prebiotics during cancer treatments. Probiotics can help restore microbial balance and have additional immunomodulatory effects [50]. For example, patients with leukemia tend to have less diverse microbiota before undergoing HSCT treatment [24] and CAR-T treatment [21]. The introduction of probiotics prior to treatment to mirror healthy individuals’ gut microbiomes could set patients up for an overall more positive outcome. It is evident that members of the phyla *Firmicutes* and *Bacteroidetes*, and likely *Verrucomicrobia*, are positively correlated with better therapeutic efficacy and decreased side effects. Probiotics could ensure a thriving bacterial population before treatment, and prebiotics could help maintain those populations throughout treatment. Current findings hint that certain bacteria are correlated with positive outcomes in patients with lymphoma [15], hematologic malignancies [21,22,25], leukemia [24], and colorectal cancer [26] using CAR-T and stem cell therapies. More research is required to establish a causal relationship between microbiota and cell-based immunotherapies. By increasing the populations of bacteria known to positively impact treatment responses, patients could experience improved therapeutic outcomes and reduced side effects post-treatment across all cell-based immunotherapy modalities.

In conclusion, the future for cell-based immunotherapies is promising. The gut microbiome plays a key role in determining treatment efficacy, mitigating adverse effects, and enhancing patient survival. As more research is conducted to understand this relationship, microbiome manipulation could become an integral part of cell-based immunotherapies, offering a pathway to more effective, personalized treatments with better patient outcomes.

## Data Availability

No new data were created or analyzed in this study.
